# Multiparameter Effect Study on Lactose and Whey Permeate Conversion to Lactic Acid and HMF Catalysed by Erbium

**DOI:** 10.3390/molecules31101596

**Published:** 2026-05-10

**Authors:** Maoline D. Houndedoke, Daniel Nickson, Michel Pouliot, Gregory S. Patience

**Affiliations:** 1 Department of Chemical Engineering, Polytechnique Montréal, Montréal, QC H3T 0A3, Canada; 2 Department of Chemical Engineering, Lancaster University, Lancaster, LA1 4YW, UK; 3 Agropur, Longueuil, QC J3Z 1G5, Canada

**Keywords:** heterogeneous catalysis, lactic acid, whey permeate, batch, scale-up

## Abstract

Making 1 kt of cheese produces 9 kt of cheese whey permeate, a waste with 5% lactose, which is either discarded or dried for animal feed. One pathway to add value to this waste is to convert it to lactic acid (LA), a monomer for polylactic acid, the largest bioplastic produced in the world. Lactose hydrolyses to glucose and galactose. While Brønsted acidity enhances lactose hydrolysis, Lewis acidity favours the formation of lactic acid. For the first time, we tested both industrial whey permeate and purified lactose as feedstocks for LA over a heterogeneous catalyst–Er_2_O_3_/Al_2_O_3_. LA Yield from whey permeate reached 14%, while the maximum yield with purified lactose was 22%. LA yield was invariant with respect to mixing speed while increasing temperature accelerates the time it takes to reach quasi-equilibrium. Yield was also independent of pressure with either air, He, N_2_, or H_2_ in the vapour space above the liquid phase in the autoclave. LA yield over spent catalyst with fresh lactose was only 11%, which indicates that the catalyst deactivates. Based on XRF analyses, the Er_2_O_3_ mass fraction dropped from 15% to 5%, with 6.4% leaching into the aqueous phase after the first step but only 0.8% after the second test.

## 1. Introduction

Lactic acid (LA) is a promising commodity as a feedstock to produce polylactic acid (PLA), a biodegradable polymer [1,2], with applications in food packaging, textiles, medication dispensing system, and cosmetics. In 2020, it represented 19% of the bioplastic market, and is projected to reach 4.5 billion USD by 2030 [3], which makes it the most produced biodegradable plastic. Anaerobic fermentation is the primary synthesis route for industrial LA [4]. Microorganisms such as *Lactobacillus plantarum* strain, and *Lactobacillus paracasei*, produce up to 0.9 g g^−1^ of lactic acid from sugar cane molasses, and 1 g g^−1^ from a mix of potato stillage waste, and sugar beet molasses [2]. A compelling feedstock for LA production is lactose derived from dairy waste streams. Lactose accounts for 5% of cheese whey permeate (CWP), a byproduct of cheese making. In 2020, cheese production in Canada reached 540 kt, which is equivalent to almost 5 Mt of CWP [5].

The reaction pathway from lactose to LA includes lactose hydrolysis to galactose and glucose, isomerisation to fructose, followed by triose formation (dihydroxyacetone, and glyceraldehyde), through retro aldol condensation, or fructose dehydration to 5-hydromethylfurfural (HMF), another green platform chemical [6,7,8]. Brønsted acidity favours lactose hydrolysis, and Lewis acidity increases selectivity towards lactic acid [9,10,11]. Saavedra and al, tested various streams of carbon sources to produce LA, including lactose, galactose, sucrose, maltose, and cane molasses [12]. Yields ranged in the following order: lactose < galactose, maltose < sucrose < sugar cane. Brinques et al. achieved a yield of more than 1 g g^−1^ of LA, starting from cheese whey as feedstock, with *Lactobacillus plantarum*, after 48 h of reaction [13]. Although fermentation processes achieve high yields, they operate with yeast extract and dilute substrates, which results in feeble volumetric productivity [1,13]. The process also requires reaction times up to 4 days, and nutrient supplement or bacteria growth [14,15,16].

An alternative is to produce LA with chemocatalysis. Wislicenus first discovered the process in 1863 [17]. Hydrogen cyanide reacts to acetaldehyde with a base catalyst to produce lactonitrile. After distillation, HCl or H_2_SO_4_ hydrolyses it to LA. However, the process depends on expensive petroleum material. Consequently, manufacturers of LA, such as Natureworks LLC (USA), and Musahino chemicals (Japan), shifted LA production to fermentation [14,18]. Few articles address the chemical conversion of lactose to lactic acid, through heterogeneous catalysis [19,20].

We previously converted lactose to LA, in 30 min, at 170 °C, with a solid catalyst made of Er impregnated on γ-Al_2_O_3_ and we proposed a catalytic mechanism for the process [11]. Here, we investigated the relationship between reactor scale, agitation, temperature, time, and fresh vs. spent catalyst on lactose conversion and LA yield. We report for the first time the heterogeneous catalysis of whey permeate to LA. CWP yield was 6% lower compared to pure lactose, potentially due to CWP ash content.

## 2. Results and Discussion

### 2.1. Internal and External Mass Transfer Limitations

**Figure 1:**
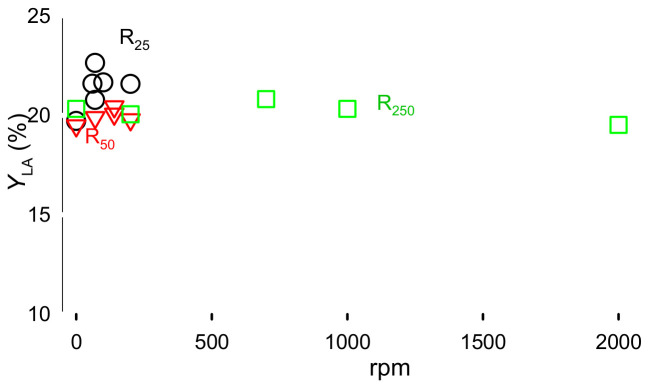
Lactic acid yield as a function of reactor volume, and magnetic stirrer rotational speed. Reaction conditions: Lactose 5%, ${\mathrm{Er}}_{15}$/γ-Al_2_O_3_, 170 °C, heating time of 1 h, with 0 to 200 rpm for $R_{25}$, 0 to 200 rpm for $R_{50}$, and 0 to 2000 rpm for $R_{250}$. The colour represents autoclave volume.

The first parameter we tested was rotational speed for different reactor volumes, as we hypothesised that stirring would enhance lactose diffusion to catalyst surface, and consequently within its pores [21]. Therefore, in case of external mass transfer limitations, lactose conversion would increase with rotation speed, and consequently LA yield. Preliminary results confirmed that external mass transfer limitation was negligible, as agitation speed slightly affected LA yield: for all conditions, the yield only varied from 20-<23% although the magnetic stirrer reached 2000 rpm in R_250_ but only 200 rpm in $R_{25}$, and $R_{50}$ (Figure 1). Mears numbers confirmed the absence of external mass transfer [22,23] (ω):

(1) $$\omega =\frac{r_{i,\mathrm{lactose}}\times \rho_{p}\times R_{p}}{k_{c}C_{i,\;\mathrm{lactose}}}<\frac{0.15}{n}$$

With:

(2) $$k_{c}=\frac{N_{\mathrm{Sh}}\times D_{m}}{2R_{p}}$$

**Figure 2:**
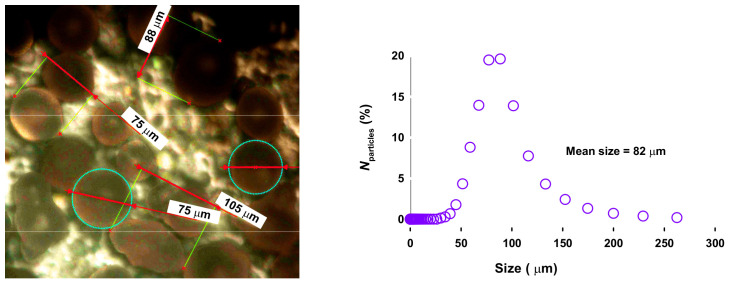
Particle size distribution of ${\mathrm{Er}}_{15}$/γ-Al_2_O_3_ (**right**), and microscope imaging. ×500 magnification (**left**).

(3) NSh=2+0.55NRe0.5NSc0.33

(4) NRe=ρ×N×D2μ

(5) $$N_{\mathrm{Sc}}=\frac{\nu }{D_{m}}$$

with, $r_{i,\mathrm{lactose}}$ the initial rate of lactose conversion, $\rho_{p}$, and $R_{p}$, the particle density furnished by the manufacturer (850 ${kg\;m}^{-3}$), and its mean radius of 82 μm (Figure 2). The mass transfer coefficient is $k_{c}$, with $D_{m}$ ($2.3\times 10^{-9}$ $m^{2}$ $s^{-1}$), the diffusion coefficient of lactose in water based on Wilke-Chang correlation [24]. As water is the main component of the liquid feed (≥95%), its viscosity at 150 °C was considered for the calculation: 181 μPa s at 150 °C [25]. $N_{\mathrm{Sh}}$ represents the Sherwood number, NRe, the Reynolds number, $N_{\mathrm{Sc}}$ as the Schmidt number, and *n*, the reaction order, considered as 1. The fluid velocity is *N* (s^−1^), *D* is the diameter of the stirrer (m) and ρ, water density (1000 kg $m^{-3}$). The kinematic density of water is ν ($m^{2}\;s$^−1^) with ν=μ/ρ. $N_{\mathrm{Sh}}$ increases by a factor of 5, from 10 to 100 mL, and consequently Mears number decreases by a factor of 5 as well (Table 1).

In all cases, the Mear number confirmed the absence of external mass transfer resistance at 150 °C, as it was lower than 0.15. To evaluate internal mass transfer, we calculated the Weisz-Prater modulus (ϕ) [16,22,26] with:

(6) $$\phi =\frac{r_{i,\mathrm{lactose}}\times \rho_{p}\times R_{p}^{2}}{D_{\mathrm{eff}}C_{i,\mathrm{lactose}}}<1$$

(7) $$D_{\mathrm{eff}}=\frac{\phi_{p}\times \sigma_{c}}{\tau }\times D_{m}$$

$D_{\mathrm{eff}}$ represents the effective diffusion coefficient;

$\phi_{p}$ is the particle porosity (0.37, calculated as the ratio of the pore volume, and total volume [11,27]);

$\sigma_{c}$ is the constriction factor, which is a function of the ratio between the maximum, and minimum pore areas, and accounts for changes in the cross sectional area of diffusion pathway; and,

τ refers to the tortuosity ($\sigma_{c}/\tau$ = 0.123) [28].

Internal mass transfer is not limiting the reaction, as ϕ was 0.2 for $R_{25}$ and 0.1 for $R_{50}$ and $R_{250}$.

### 2.2. Temperature

Lactose reacted at 150 °C, 170 °C, and 190 °C with ${\mathrm{Er}}_{15}$/γ-Al_2_O_3_, from 30 min–2 h. We compared previously reported data in $R_{25}$ [11] with $R_{50}$, and $R_{250}$.

**Figure 3:**
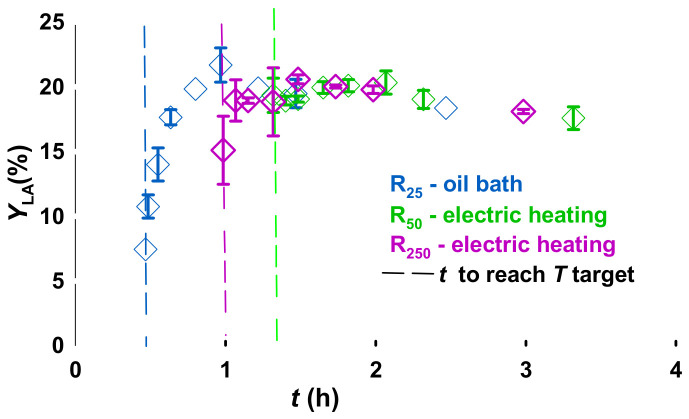
Lactic acid yield in function of reactor volume, and time. Reaction conditions: Lactose 5%, ${\mathrm{Er}}_{15}$/γ-Al_2_O_3_, 170 °C, 70 rpm for $R_{25}$ [11], 140 rpm for $R_{50}$, and 700 rpm for $R_{250}$. Error bars represent standard deviation with n=2. The vertical, coloured-dashed line represents the time it took the autoclave to reach the set-point temperature.

**Figure 4:**
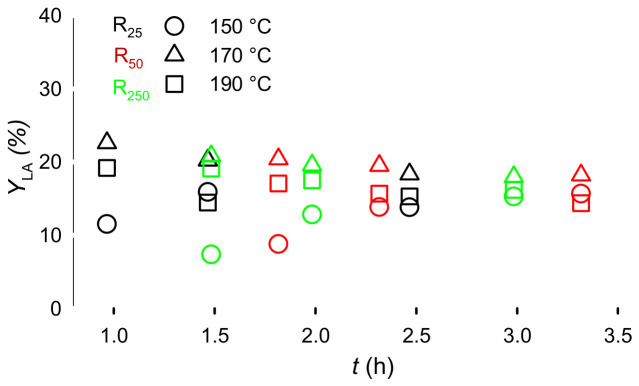
Lactic acid yield as a function of reactor volume, temperature, and heating time. Reaction conditions: Lactose 5%, ${\mathrm{Er}}_{15}$/γ-Al_2_O_3_, 70 rpm for $R_{25}$ [11], 140 rpm for $R_{50}$, and 700 rpm for $R_{250}$. The colour represents autoclave volume, while the symbol type corresponds to temperature. It took 30 min to heat $R_{25}$ (black symbols) from ambient to the set-point. The time on the *y*-axis includes the heating time and the time at the set temperature.

While the autoclave heats, lactose reacts (Figure 3). So, selecting a time zero is somewhat arbitrary: For $R_{250}$, it takes 63 min to reach 150 °C and another 10 min to reach 190 °C. To account for the reaction during the heating period, we considered the time to reach the target temperature and the reaction time as heating time (Figure 4). At 190 °C, the yield is initially high as the lactose reacts during the transient heating and then drops as lactic acid began to degrade. This trend is repeated at 170 °C. From 2 h of reaction time (corresponding to 2.3 h of heating time), LA yield is essentially independent of volume or temperature.

Here, we are the first to report that as reaction time increases, LA yield sourced from lactose, reaches a quasi-equilibrium independent of conditions. Bicker et al. reacted dihydroxyacetone, fructose, and glucose over ZnSO_4_ in a continuous reactor at subcritical conditions (200–360 °C and 25 MPa) [29]. At residence times above 100 s, conversion of the substrates was essentially 100%. Selectivity with glucose as a feedstock was 24% while it reached 28% with fructose (hexoses) and 55% for the dihydroxyacetone (3 carbon molecule) (Figures 8–10 in their article). For each reaction, the exit concentration of the lactic acid was close to 50% of the inlet substrate concentration. Lactic acid yield from fructose over erbium was equivalent to lactose (25% versus 23%), which is similar to the results of Bicker et al. but at very different conditions [11,29].

Wang et al. converted glucose, fructose, mannose, and sucrose to ethyl lactate in ethanol. The conversion involves a retro-aldol condensation of fructose to trioses (glyceraldehyde, and dihydroxyacetone), followed by their dehydration to methylglyoxal, and acetalisation to yield ethyl lactate (EL) [22,30]. As the retro-aldol reaction is endothermic, increasing temperature thermodynamically benefited EL production, which confirms the observed behaviour in this study.

### 2.3. Time

A complementary study evaluated the impact of volume and time on LA yield at 170 °C. The $R_{50}$ and $R_{250}$ curves in Figure 3 overlap sooner than $R_{25}$. After 1 h of heating, $R_{25}$ reached a yield of 23%, while it was only 17% in the $R_{250}$.

**Figure 5:**
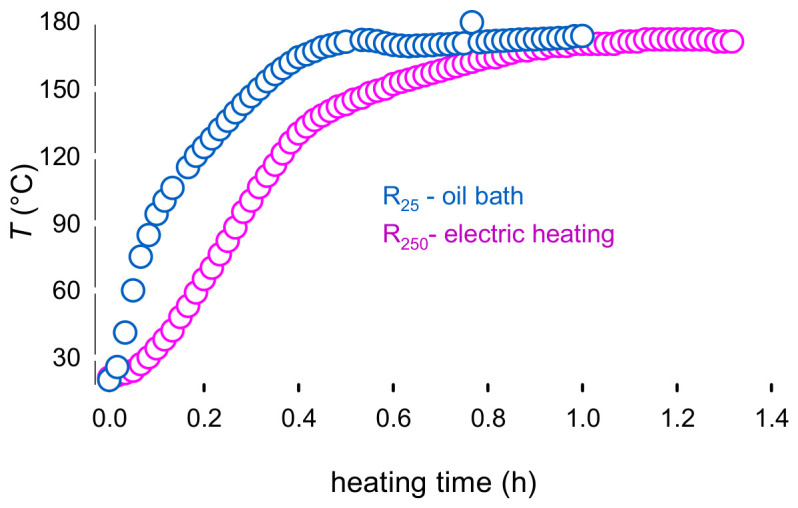
Heating curves for $R_{25}$ and $R_{250}$. The target temperature was 170 °C. Reaction conditions: Lactose 5%, ${\mathrm{Er}}_{15}$/γ-Al_2_O_3_, 70 rpm for $R_{25}$, and 700 rpm for $R_{250}$.

This trend highlights the differences in the heating rates of the reactors: An electric mantle heated $R_{50}$ and $R_{250}$ autoclaves, an oil bath heated the $R_{25}$ vessel: $R_{25}$ heated at 6 ° $\min^{-1}$ while $R_{250}$ heated at 3 ° $\min^{-1}$ (Figure 5). Theses values match the heating rate range in the literature for subcritical water processes, from 2 to 7 °$\min^{-1}$ [31,32]. Consequently, the initial time of reaction for R1 ($t_{0,R_{25}}$) started after 29 min of heating, while $t_{0,R_{250}}$, was after 59 min. This is the first report of heating time effect on the multiscale conversion of lactose, with a heterogeneous catalyst. An analogous process of brewer’s spent grain conversion to glucose, xylose, and arabinose by Alonso et al., confirmed that heating time was a critical factor. Laboratory scale experiments with a heating time of 16 min yielded a higher arabinose content than at the pilot scale, with a heating time of 5 min. However, both were compared at 22 min, not accounting for the time delay induced by the heating time, which explains the discrepancies in their results [32]. The difference becomes insignificant after 1.5 h (Figure 3).

### 2.4. Pressure

**Figure 6:**
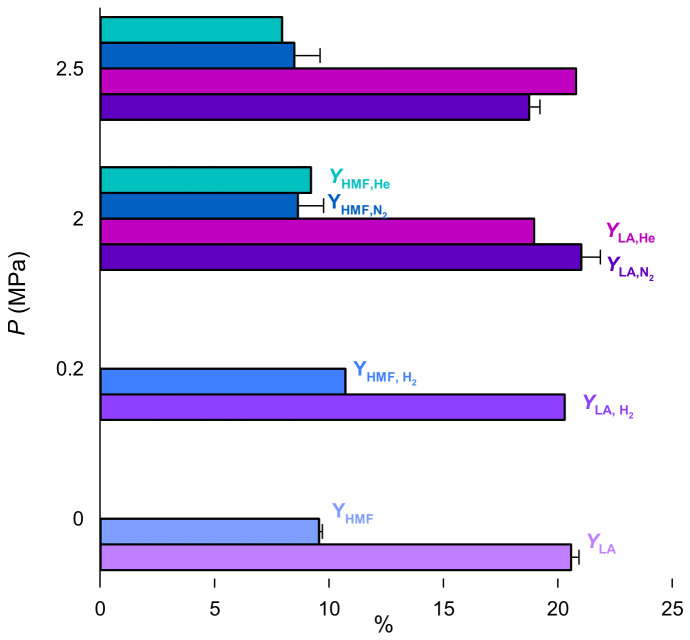
Influence of nitrogen and helium on lactose conversion, lactic acid, and HMF yield. Reaction conditions: Lactose 5%, ${\mathrm{Er}}_{15}$/γ-Al_2_O_3_, 170 °C, $R_{250}$ at 700 rpm. Error bars represent standard deviation with *n* = 2.

The influence of pressure on lactose conversion, LA, and HMF yield was assessed inside $R_{250}$ (Figure 6) at 3 levels: ambient, 2 MPa, and 2.5 MPa. We also tested 0.2 MPa to assess the impact of H_2_ on LA yield. LA reached a maximum yield of 22% at 2.5 MPa with N_2_. With He and H_2_, LA yield was essentially constant from 0.2 MPa to 2.5 MPa. Lactose conversion and HMF yield are also quite constant for both He and N_2_, ranging from 95% to 98%, and 9% to 10%. With H_2_, HMF reached 11%, while lactose conversion was 95%.

An analysis of variance (ANOVA) assessed if the yields were sensitive to pressure in the presence of N_2_. The *p*-value > 0.05, which suggests that LA yield is insensitive to $P_{N2}$. Monteiro et al. performed the hydrothermal treatment of sugarcane bagasse at 2.5 MPa and 10 MPa. Their study aligns with our results as they observed that pressure was insignificant for oligosaccharides release [33].

### 2.5. Coked Catalyst Activity

**Figure 7:**
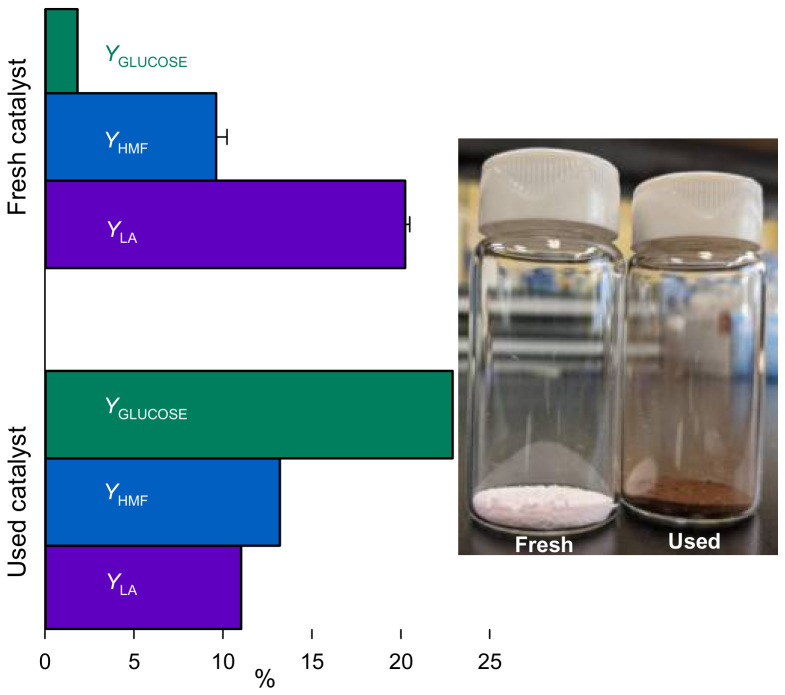
Used catalyst activity for lactose, and whey permeate conversion to lactic acid, HMF, and glucose. Reaction conditions: Lactose 5%, 30 min. ${\mathrm{Er}}_{15}$/γ-Al_2_O_3_, 170 °C, $R_{50}$ at 140 rpm. Experiment with fresh catalyst repeated twice. Error bars represent standard deviation with n=2.

As previous experimentation showed that LA yield increased with temperature and time until reaching an equilibrium at a complete conversion of lactose, we hypothesised that ${\mathrm{Er}}_{15}$/γ-Al_2_O_3_ was still active. To test this assumption, spent catalyst reacted with fresh lactose (Figure 7). Surprisingly, the lactose conversion reached 95% with a LA yield of 11% and 13% HMF yield. When compared to fresh catalyst, lactic acid yield decreased by 8% (absolute), but HMF increased by 3%. While used catalyst still hydrolyses lactose, the consecutive conversion of monosaccharides is challenging as the yield to glucose reached 22%. We compared the XRF analysis of the fresh catalyst with the spent one. Er leached during the reaction, as it decreased from 15% to 5%. The analysis of the liquid product confirmed the presence of Er in the sample (Table 2).

Er can replace hydrogen on Al-OH surface bonds, decreasing Brønsted acidity. With Er leaching, the reverse effect may increase Brønsted acidity, which favours lactose hydrolysis and fructose dehydration to HMF [10,34]. Interestingly, only 0.8% of the erbium leached into the liquid sample compared to 6.4% with fresh catalyst. Amorphous carbon deposited on the catalyst surface may encapsulate Er, which decreases leaching [35]. Er is strongly oxophilic and may bind preferentially with oxygenated compounds of the reaction products than the catalyst [36,37].

An XPS analysis of the spent catalyst characterised the chemical state of C, Al, and O at the surface of the catalyst (Table 3).

**Figure 8:**
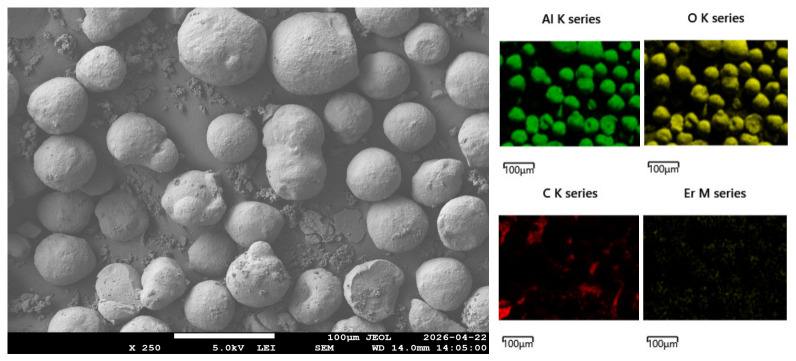
SEM-EDX of the spent catalyst. ×250 magnification.

C-C and C=C bonds represented 54% of the carbon quantified by XPS. The analysis also confirmed the presence of surface AlOH. SEM revealed that the formed carbon exists mainly in the form of aggregates, not bound to the support (Figure 8).

### 2.6. Towards Whey Permeate Conversion

**Figure 9:**
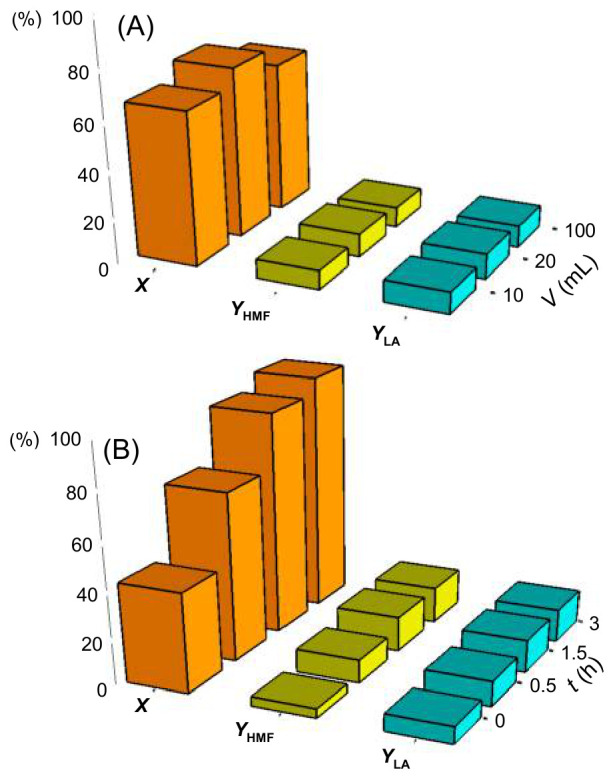
Influence of reaction volume (**A**) and time (**B**) on whey permeate conversion (*X*), lactic acid ($Y_{LA}$), and HMF yield ($Y_{HMF}$). Reaction conditions: whey permeate 4.4%, ${\mathrm{Er}}_{15}$/γ-Al_2_O_3_, 170 °C, 70 rpm for $R_{25}\;$ [11], 140 rpm for $R_{50}$, and 700 rpm for $R_{250}$. The results in (**B**) are the mean values of two tests at 170 °C and 30 min.

Wet tested industrial whey permeate with the three autoclaves and varying reaction time from 0 to 3 h. The LA yield was highest in $R_{25}$ at 11%, while the HMF yield was 10%, and the whey permeate conversion was 71% (Figure 9A). Subsequent testing consisted of varying the reaction time in $R_{25}$. Conversion, LA, and HMF yield increased with time up to 1.5 h, with respective percentages of 93%, 14%, and 15%. Longer reaction time (3 h) increased whey permeate conversion to 99%, while LA, and HMF yield remained constant (Figure 9B). This is the first record of heterogeneous conversion of whey permeate to LA, with a solid catalyst, which represents a proof of concept. These results agree with Turner et al., who performed the fermentation of lactose, and whey permeate to LA with a yeast engineered *S. cerevisiae*. With lactose, LA yield reached 0.58 g g−1 in 143 h, while they obtained 0.358 g g−1 with whey permeate in 110 h [38]. CWP furnished by Agropur contains 6.5% of ash [39], which may decrease lactose hydrolysis.

## 3. Materials and Methods

### 3.1. Whey Permeate Powder Preparation

The conventional process for ultra-filtration (UF) of whey permeate powders involves three steps: solids concentration, lactose crystallisation, and spray drying. First, UF membranes recover proteins from cheese whey with a molecular weight cut-off of 10,000 Da. The liquid permeate generated contains 4.6–4.9% of lactose and less than 1% ash, vitamins, and other nitrogenous compounds, such as urea.

The concentration step starts with vacuum evaporation in 7 multi-effect evaporators. From one effect to the other one, the temperature ranges from 65 to 80 °C; the solids concentration gradually increases to reach 60% solids in the final effect, which also serves to cool the product to below 40 °C. The concentrated liquid is transferred to crystallisers: stirred tanks that gradually reduce the temperature to below 10 °C over 8 to 16 h. This step allows the soluble lactose to crystallise into alpha-lactose monohydrate crystals, which is less hygroscopic and much easier to dry. The powders will also be more resistant to caking and moisture absorption during storage.

The final step consists of pumping lactose crystal slurry into a spray dryer, which reduces the moisture content to approximately 4% (Table 4). This involves atomising the crystal suspension into a fine mist with high-pressure jets at the top of the tower. A supply of hot air evaporates the water, and fine particles are collected at the bottom of the tower; the drying air carries product dust, which is separated by cyclones before being filtered and released into the atmosphere. The collected fine particles are added to those recovered at the bottom of the tower. The product is then stored in a silo until packaging.

### 3.2. Catalyst Synthesis

We reported the catalyst synthesis process in a previous study [11]: 2 g of Al_2_O_3_ (Puralox SCCa-5/200, Sasol Chemicals, Brunsbüttel, Germany) was combined with 0.62 g of 99% ErCl_3_·6H_2_O (Sigma Aldrich, Oakville, ON, Canada) in 1 g of water at room temperature. The powder was dried in an oven overnight at 60 °C, then calcined for 4 h at 550 °C, with a ramp of 5 °Cmin^−1^. This treatment oxidized the Er while driving off most of the Cl so that the catalyst retained a mass fraction of 0.15 g g^−1^ Er_2_O_3_. We adopted the following nomenclature to represent the catalyst: ${\mathrm{Er}}_{15}$/Al_2_O_3_.

### 3.3. Batch Experiments

**Figure 10:**
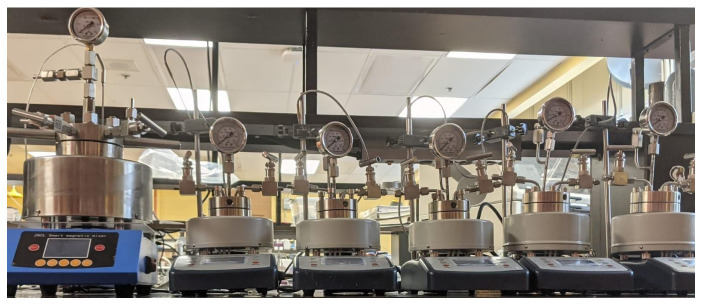
Multi scale lab reactors. From left to right: 250 mL ($R_{250}$), 3 × 25 mL ($R_{25}$), and 2 × 50 mL ($R_{50}$).

Stainless steel autoclaves (SS304L) with volumes of 50 mL ($R_{50}$) and 250 mL ($R_{250}$) (Figure 10), were charged with 20 mL and 100 mL of a 5% lactose solution with 0.8 g and 4 g of ${\mathrm{Er}}_{15}$/Al_2_O_3_. After sealing the reactors, an electric mantle heated the solution from ambient temperature to the set-point (150–190 °C). After the autoclave reached the set-point, it was held at that temperature for 30 min to 2 h. Complementary experiments were completed at 170 °C in which we examined the solution after it reached the set-point and at 5 min, 10 min, 20 min, 30 min, 45 min, and 1 h. The results were compared to previously reported data in a 25 mL batch autoclave ($R_{25}$ filled with 10 mL of lactose) [11]. To evaluate the effect of pressure, we blanketed $R_{250}$ with N_2_, He, or H_2_, at 0.2 MPa, 2 MPa, and 2.5 MPa. He has a higher thermal conductivity than N_2_, and H_2_ can act as a reducing agent of coke precursors [40,41,42]. Spray-dried cheese whey permeate (Agropur) reacted with ${\mathrm{Er}}_{15}$/Al_2_O_3_ in water in the $R_{25}$ and $R_{250}$ autoclaves. To assess time influence on conversion and yield, we varied the reaction time from 0 to 3 h, at 170 °C.

In all the experiments, we plunged the autoclave into an ice bath to quench the reaction as soon as possible. A Büchner funnel separated the liquid product from the spent catalyst. Distilled water washed the catalyst, and a 0.22 μm polytetrafluoroethylene membrane filtered the samples prior to high performance liquid chromatography (HPLC) analyses.

### 3.4. Products Quantification

An HPLC (UHPLC 3000, with UV, and RI detectors, Thermo Fisher Scientific, Oakville, ON, Canada) quantified reaction products, based on calibration curves for lactose, lactic acid, and HMF. The liquid product eluted in a Aminex HPX-87H column (Agilent Technologies Canada Inc., Mississauga, ON, Canada), with 0.05 N H_2_SO_4_ as solvent. Conversion and carbon yield are defined with Equations (8)–(10), where *n* represents moles:

(8) $$X=\left(\frac{n_{i,\mathrm{lactose}}-n_{f,\mathrm{lactose}}}{n_{i,\mathrm{lactose}}}\right)$$

(9) $$Y_{\mathrm{LA}}=\left(\frac{3}{12}\right)\times \left(\frac{n_{\mathrm{LA}}}{n_{\mathrm{lactose}}}\right)$$

(10) $$Y_{\mathrm{HMF}}=\left(\frac{6}{12}\right)\times \left(\frac{n_{\mathrm{HMF}}}{n_{\mathrm{lactose}}}\right)$$

### 3.5. Characterisation and Statistical Analysis

An Epsilon 4 X-Ray fluorescence—XRF (Malvern PANalytical, Montreal, QC, Canada), equipped with a silicon drift detector at 135 eV, measured the bulk element composition of the spent catalyst [43]. An Horiba LA-950 particle size analyser (HORIBA) measured the mean size of ${\mathrm{Er}}_{15}$/Al_2_O_3_ [44] and a Keyence microscope (Keyence, Mississauga, Canada) captured imaging of the particles with their diameter, at 500× magnification. An analysis of variance evaluated significant differences for reactions supplemented with N_2_ [45] with Statistica (Version 14.0.1.25, TIBCO Software, San Ramon, CA, USA). Analyses were performed by selecting a confidence level of 95% on Statistica. X-ray spectroscopy (XPS) of the spent catalyst, with a Thermo Scientific ESCALAB 250Xi analyzer (Thermo Fisher Scientific, Canada) identified the elements chemical state and quantified them, with an Al Kα monochromatic source at 218.8 W (14.7 kV, and 14.9 mA). The pass energy applied to the survey spectra was 150 eV, with a step size of 1 eV. A JEOL JSM-7600F scanning electron microscope (JEOL USA, Inc., Peabody, MA, USA) revealed the morphology of the spent catalyst (resolution of 1 nm at 15 kV) and the surface element composition through EDX.

## 4. Conclusions

Here, we are the first to report the impact of mixing, temperature, time, and pressure on heterogeneous lactose conversion to LA, in multiscale-reactors. For the tested reaction volumes, LA yield was independent of agitation speed, and it reached an equilibrium when increasing temperature, and heating time. Similarly to mixing, ANOVA results confirmed that yield was independent of pressure, and gas composition (H_2_, N_2_ or He). Lactose reaction with spent catalyst suggests that carbon deposited on spent ${\mathrm{Er}}_{15}$/γ-Al_2_O_3_ may encapsulate Er, which explains the lower leaching effect at the consecutive second use of the catalyst. XPS revealed that the carbon chemical state is mainly C-C and C=C type bonds, while SEM-EDX indicate that it forms aggregates, not bound to the support of the catalyst.

Testing of whey permeate resulted in a maximum yield of 14% LA and 15% of HMF. These results provide valuable insights for dairy whey permeate valourisation to lactic acid. Future research should focus on enhancing the strength of Er links to the support and on adequate carbon treatment to conserve the properties of the catalyst.

## Figures and Tables

**Table 1:** Mears parameters for multiscale lactose conversion.

Volume mL	*N*_Sh_	*k*_c_ m^2^s^−1^	$r_{i}$, Lactose mol ^2^s^−1^kg^−1^	Mears Number
10	108	3 × 10^−3^	2 × 10^−3^	1 × 10^−4^
20	152	4 × 10^−3^	1 × 10^−3^	7 × 10^−5^
100	507	1 × 10^−2^	1 × 10^−3^	2 × 10^−5^

**Table 2:** XRF analysis of fresh and spent catalyst.

Sample	Er_2_O_3_ (%)	
	**Fresh Catalyst**	**Used Catalyst**
Powder	15	5
Liquid sample	6	0.7
Liquid sample (wash)	0.4	0.1

**Table 3:** XPS Analysis of spent catalyst.

Element	Chemical State	Peak Binding Energy (eV)	Atomic %
C	C-C, C=C	284.8	17.8
C	C-O	286.3	8.3
C	C=O	287.6	3.8
C	O-C=O	289.1	2.5
C	π to π* (aromatic)	291.5	0.6
Al	Al_2_O_3_	74.9	16.8
O	Al_2_O_3_	531.05	11.2
O	C=O	532.5	7.9
O	C-O, C-O-C	533.1	9.4
O	O *-C=O (carboxylic)	534.2	2.8
O	Al-OH	531.9	18.9

**Table 4:** UF whey permeate powder composition [39].

%	Typical Values	Guaranteed Content
Ash	7	max 8
Lactose (by difference)	87	82–92
Moisture	4	Max 5
Salt	1	Max 3

## Data Availability

The original contributions presented in this study are included in the article. Further inquiries can be directed to the corresponding author.
